# Patient’s view on: “Low-protein diet with personalized support in advanced chronic kidney disease: association with disease progression, dialysis delay, and mortality”

**DOI:** 10.1093/ckj/sfaf385

**Published:** 2025-12-09

**Authors:** Takamasa Iwakura

**Affiliations:** Preventive Medicine for Severe Progression of Lifestyle-Related Diseases, Hamamatsu University School of Medicine, Hamamatsu, Japan; First department of Medicine, Division of Nephrology, Hamamatsu University School of Medicine, Hamamatsu, Japan

This observational study from Bologna, Italy, evaluating a moderately restricted low-protein diet (LPD) in patients with advanced CKD, was highly engaging from a patient representative’s perspective. It was particularly meaningful that the authors focused not on adding another medication, but on nutritional management, an area where patients can actively participate in their care.

Patients with CKD often take multiple medications and may be reluctant to add yet another drug. In contrast, dietary modification can be more acceptable because it allows individuals to feel involved in their treatment decisions. At the same time, dietary restrictions directly affect daily life, and therefore quality of life (QOL) concerns are just as important as adherence. Historically, strict protein restriction has suffered from poor adherence and significant lifestyle burden [1]. In this context, the moderate restriction examined in this study (0.6–0.7 g/kg/day), combined with personalized support and a weekly unrestricted meal, seems far more feasible. The high acceptance (96%) and moderate adherence (about 60%) underscore the practicality of such an approach.

Several aspects of the study population are noteworthy. The participants had relatively high-risk characteristics, including a median proteinuria of 0.94 g per 24 hours, hypertension in nearly 87%, and diabetes in 43%, yet the use of renin–angiotensin–aldosterone system (RAAS) inhibitors, a cornerstone of CKD treatment, was unexpectedly low at ∼30%. This finding could not be explained by hyperkalemia or metabolic acidosis, and the use of potassium binders was also limited. Because CKD progresses slowly, it is critically important to optimize fundamental therapeutic measures before patients reach advanced stages. These measures include adequate blood pressure control and appropriate use of RAAS inhibitors, and early intervention is expected to maximize the potential for slowing kidney function decline.

In this study, conducted between 2021 and 2023, the mean systolic blood pressure at enrollment was well above the KDIGO 2021 recommended target of <120 mmHg [2], suggesting that treatment may not have been fully optimized before nephrology referral. In addition, many older patients may have found it difficult to reach lower blood pressure targets due to medication tolerability issues. Differences in recommended blood pressure targets for older adults across international guidelines may also help explain why blood pressure tended to remain higher in this cohort. Furthermore, the low use of RAAS inhibitors may have contributed to suboptimal blood pressure control. At the last visit, blood pressure was still not sufficiently controlled, and discontinuation of RAAS inhibitors was relatively common. Given these circumstances, the effects of the LPD should be interpreted in the context of suboptimal baseline management. Even so, the improvements observed after the introduction of the LPD suggest that individualized nutritional intervention can play a meaningful role in supporting kidney health.

It is also noteworthy that systolic blood pressure in the LPD group was higher than in the control group at baseline but decreased to comparable levels by the last visit. This raises the possibility that part of the observed improvement in kidney function may have been influenced by reductions in intraglomerular pressure resulting from better blood pressure control.

The study period (2021–2023) coincided with the early adoption of sodium-glucose cotransporter 2 (SGLT2) inhibitors in CKD therapy, and determining how best to integrate these agents with dietary interventions remains an important area for future investigation. Moreover, given the timing of the study, it is possible that some patients initiated SGLT2 inhibitors during follow-up, and such exposure may have partially contributed to the renal function changes observed in this cohort. In the authors’ interaction analysis, the benefits of the LPD were more pronounced in patients not receiving RAAS inhibitors. This may reflect the fact that both LPD and RAAS inhibitors share the mechanism of lowering intraglomerular pressure, thereby reducing the likelihood of additive effects when used together. Furthermore, because the renoprotective effects of SGLT2 inhibitors are also frequently attributed to improvements in intraglomerular pressure, more precise future studies are needed to determine whether combining SGLT2 inhibitors with an LPD yields synergistic or additive benefits.

A particularly striking finding was the annual eGFR slope improvement of −1.76 ml/min/1.73 m² per year in the LPD group compared with controls. This degree of improvement is comparable to, and does not appear inferior to, the slope changes reported in major SGLT2 inhibitor trials such as DAPA-CKD [3] and EMPA-KIDNEY [4], as well as the prespecified subgroup analysis from DAPA-CKD [5]. However, caution is needed when interpreting this difference. Beyond the low use of RAAS inhibitors in the present cohort, the studies differ fundamentally in design: this being a real-world observational study, whereas DAPA-CKD and EMPA-KIDNEY were rigorously conducted randomized controlled trials. These distinctions in baseline therapy, population characteristics, and methodological rigor limit direct comparability. Even so, the findings highlight the potential renal impact of sustained nutritional intervention.

Of course, patients who chose to follow the LPD may inherently have higher adherence to medical advice and healthier lifestyle patterns overall. Nevertheless, this study clearly demonstrates the value of personalized nutritional support and suggests that patient empowerment, enabling individuals to decide whether to incorporate an LPD into their care plan, is central to successful implementation.

In conclusion, this study suggests that a moderate, individualized LPD is both feasible and potentially beneficial in advanced CKD. Further research should clarify how dietary interventions can best be integrated with contemporary CKD therapies, assess their long-term sustainability, and identify approaches to support patients in maintaining not only adherence but also QOL, an essential component of patient-centered care.
